# Vascular endothelial growth factor and acute mountain sickness

**DOI:** 10.4103/0974-2700.44675

**Published:** 2009

**Authors:** Eric Nilles, Helen Sayward, Gail D'Onofrio

**Affiliations:** Department of Emergency Medicine, University of Iowa, Iowa City IA 52242-1009, USA; 1 Department of Surgery, Section of Emergency Medicine, Yale University School of Medicine, 64 Congress Avenue, Suite 260, New Haven, CT 06519-1315, USA

**Keywords:** Acute mountain sickness, high altitude illness, vascular endothelial growth factor

## Abstract

**Study Objective:**

Despite causing significant morbidity throughout the mountainous regions of the world, the pathophysiology of acute mountain sickness (AMS) remains poorly understood. This study aims to improve the understanding of altitude illness by determining if vascular endothelial growth factor (VEGF) plays a role in the development of AMS. The purpose of this study was to determine if elevated plasma VEGF correlates with increased symptoms of AMS at high altitude.

**Patients and Methods:**

This is a prospective study of a cohort of healthy climbers on Denali (Mount McKinley) in Alaska at 14, 200 feet. Baseline demographics, medications, rates of ascent, and AMS scores were recorded. Pulse oximetry measurements and venous blood samples were obtained. Comparisons were made between mountaineers with and without AMS.

**Results:**

Seventy-two climbers were approached for participation in the study; 21 (29%) refused. Of the 51 climbers participating in the study, 14 subjects (27.5%) had symptoms of AMS and 37 subjects (72.5%) were free of symptoms of AMS. Plasma VEGF levels were 79.14 pg/dl (SD: 121.44) and 57.57pg/dl (SD: 102.71) in the AMS and non-AMS groups, respectively. These results were nonsignificant. Similarly, comparison of sex, age, rate of ascent, pulse oximetry values, or history of altitude illness did not reveal significant differences between the AMS and non-AMS groups.

**Conclusion:**

This study does not provide evidence in support of the theory that plasma VEGF correlates with symptoms of AMS.

## INTRODUCTION

During ascent to high altitudes, climbers, skiers, trekkers, and other outdoor enthusiasts experience a variety of high altitude illnesses. These include acute mountain sickness (AMS), high-altitude cerebral edema (HACE), and high-altitude pulmonary edema (HAPE). By far the most common of these conditions, AMS is a clinical syndrome characterized by headache, nausea, vomiting, lethargy, irritability, and sleep disturbances; on occasion, it leads on to HACE. Although the diagnosis, treatment, and prevention of this illness are quite well described, the pathophysiology of AMS remains poorly understood.[1]

HACE and AMS are closely related illnesses, with HACE being the clinical and pathophysiologic end-stage of AMS. Like the initial stages of HACE, moderate and severe AMS is characterized by progressive vasogenic cerebral edema.[2] This is an important distinction, for while vasogenic cerebral edema is secondary to capillary leak, cytotoxic (intracellular) cerebral edema is secondary to cell injury and death. Cerebral hemodynamic dysregulation, leading to increased cerebral capillary pressures and depression of normal cerebral vascular auto-regulation, likely plays an important role in the development of cerebral edema.[3] However, these factors alone are not considered sufficient,[4] and additional neurohumoral mediators are believed to play a prominent role in the development of increased capillary permeability.[1] Speculation has focused on several possible mediators, most notably inducible nitric oxide synthase (iNOS) and vascular endothelial growth factor (VEGF). This study focuses on the role of VEGF in AMS.

VEGF, previously known as vascular permeability factor, is an endothelial-specific mitogen essential for the process of angiogenesis. VEGF exerts its effect by disrupting basement membrane ligans, thus increasing capillary permeability and leading to plasma leak from capillaries.[5] As has been documented widely in the literature, animal and *in vitro* studies demonstrate a consistent up-regulation of VEGF in endothelial tissues that are subject to hypoxic conditions.[6–8] Also of interest is the finding that corticosteroids, which are potent down-regulators of VEGF expression *in vitro*,[9] are highly effective in the prevention and treatment of AMS and HACE.[10] The aim of this study was to determine if VEGF levels at high altitude are increased in individuals with symptoms of AMS as compared to individuals without AMS.

## PATIENTS AND METHODS

### Study design

This was an observational, prospective cohort study utilizing a convenience sample of subjects. The study was conducted from May 23^rd^ to June 23^rd^, 2002. The Institutional Review Board (IRB) of Yale University approved the study. The United States Department of the Interior and the National Park Service authorized a scientific research permit.

### Study setting and participants

Climbers were invited to participate in the study while acclimatizing at the 14,200-foot camp on the West Buttress route of Denali (Mount McKinley, Alaska). All climbers were on their way up the mountain and had been acclimatizing at this elevation for a minimum of 24 h to avoid confusing symptoms of AMS with that of dehydration or exhaustion. No climbers descending the mountain were included in the study. All subjects signed IRB-approved consent forms. Exclusion criteria included self-reported pregnancy, concurrent illness (other than altitude-related illnesses), age less that 18 years, and recent corticosteroid use.

### Study protocol

Subjects provided demographic data and completed a standard Lake Louise AMS score worksheet as described by the Lake Louise consensus on the definition and quantification of altitude illness.[11] Ascent profile, i.e., the time taken for ascent from the 7,200-feet base camp to 14,200 feet, was self-reported. Oxygen saturation was determined using a portable pulse oximeter. Each subject provided 5 ml of venous blood, which was collected in citrate-containing phlebotomy tubes. Using a National Park Service (NPS) solar energy source, which could only be utilized during sunny weather, the samples were centrifuged at 3000g for 10 min. The serum supernatant was decanted into clean phlebotomy tubes and kept frozen. Plasma VEGF, rather than serum VEGF, was measured to avoid cross-contamination with leukocyte or platelet VEGF.[12] The samples were kept frozen until analysis in New Haven, CT. Free plasma VEGF levels were determined using a standard commercial VEGF enzyme-linked immunosorbent assay (R and D Systems Inc., Minneapolis, MN, USA). All sample assays were run in duplicate.

### Statistical analysis

SPSS, version 12.0, was used for data analysis. Categorical data were compared using chi-square analysis. Continuous data means were compared using independent samples t-tests. *P*-values less than 0.05 were considered statistically significant.

## RESULTS

There were 51 subjects enrolled in the study, 14 with AMS and 37 without AMS. Twenty-one climbers refused enrollment. The reasons for refusal were twofold: dislike of venipuncture and concerns regarding subsequent decreased oxygen-carrying capacity. The mean age, sex, rate of ascent, incidence of prior high altitude illnesses, and plasma VEGF levels are presented [Table 1]. The 95% confidence limits for the difference in mean VEGF levels between the AMS and non-AMS groups were not statistically significant. Similarly, none of the other measured variables including sex, age, rate of ascent, oxygen saturation, or history of previous symptoms were significantly different between the AMS and non-AMS groups

**Table 1:** Demographics and data analysis of subjects

	AMS n=14	Non-AMS n=37	Significance
Sex n (%)			
Females	1 (7.1)	8 (21.6)	ns
Males	13 (92.9)	29 (78.4)	ns
Age m years (sd)	34.4 (7.29)	32.9 (8.51)	ns
Rate of Ascent m days (sd)	7.57 (2.43)	8.68 (2.64)	ns
Pulse Oximetry m % (sd)	83.3 (4.54)	85.8 (3.59)	ns
Previous Sx n (%)	8 (57.2)	11 (29.7)	ns
VEFG m pg/mL (sd)	79.14 (121.44)	57.57 (102.71)	ns

We also did a review of subjects taking prophylactic acetazolamide (*n* = 5), but this did not reveal a statistically significant difference in their plasma VEGF levels. Twenty percent (1/5 subjects) of these climbers developed symptoms of AMS. Similarly, subjects taking prophylactic ginkgo biloba (*n* = 3) did not reveal any significant difference in their plasma VEGF levels. Sixty-six percent (2/3 subjects) had symptoms of AMS although, clearly, little generalization can be made.

## DISCUSSION

This study fails to identify an increase in plasma VEGF levels in subjects with AMS; this finding echoes several other small studies that reached similar conclusions.[14–16] These human studies conducted at high altitudes have failed to reach a consensus regarding the role of VEGF in AMS and, further, whether VEGF levels do actually increase at high altitude. Maloney *et al*. showed a trend toward decreased plasma VEGF levels at high altitude, but they did not document any significant difference in VEGF levels between subjects with and without AMS. Gunga *et al*. showed a significant decrease in plasma VEGF levels in marathon runners following a high-altitude marathon to an elevation of 4722 meters. This study did not evaluate for presence of AMS. Walter *et al*. found a significant increase in both arterial and mixed venous VEGF levels in 14 subjects ascending to an elevation of 4,559 meters. However, there was no significant difference in VEGF levels between the AMS and non-AMS groups. In summary, despite the mixed results regarding changes in plasma VEGF levels at high altitude, no study has documented a statistically significant correlation between AMS and elevated plasma VEGF, the primary point of interest in this study.

These reports are of interest given the otherwise robust evidence supporting the theories that hypoxia stimulates VEGF production and, further, that VEGF is a powerful mediator of capillary permeability. Possible explanations for the contradictory nature of these findings include, most notably, the questionable validity of the assumption that peripheral (or even central) plasma VEGF levels reflect local intracerebral VEGF concentrations. As was elegantly determined by Walter *et al*., circulating VEGF levels can be highly variable depending upon the vascular site from which blood was obtained, with arterial blood having consistently higher levels than either mixed venous or capillary samples. Thus, a reasonable question would be whether cerebrospinal fluid (CSF) would better reflect local intracerebral VEGF concentrations; however, there are obvious difficulties associated with conducting a study to determine this in humans. The pre-study assumption of this author was that despite the possibility of a poor correlation between intracerebral VEGF and serum VEGF concentrations, a discernable and consistent relationship may exist and would provide supporting evidence for a role of VEGF in AMS (and thus, presumably, in HACE). A single previous study supports the assumption that local tissue and circulating VEGF levels are correlated[17]; it is questionable, however, whether these findings could be extrapolated to the central nervous system.

Another possible, and interesting, explanation for the lack of *in vivo* hypoxia-induced plasma VEGF production is based on several studies that indicate that acidosis plays a role in the stimulation of VEGF synthesis and expression.[18–21] In fact, these studies promote the theory that acidosis and hypoxia independently up-regulate VEGF production. Given that hyperventilation, and the subsequent respiratory alkalosis, is an integral part of human high-altitude physiology, one may speculate on an inhibitory role of alkalemia on VEGF expression. Examining venous pH in concert with serum VEGF may shed light on the role alkalemia plays in the *in vivo* production of circulating VEGF. Of course, this would not answer the question of whether local, intracerebral pH levels are significantly different from the serum pH and whether local intracranial VEGF production is significantly different from systemic production.

The majority of the VEGF-related literature provides promising theoretical evidence for a role of VEGF in moderate to severe AMS and HACE. However, the few high-altitude human studies that have been conducted have failed to support this assumption. Certainly, this may be due to the fact that VEGF does not, in fact, play a role in the pathophysiological changes in AMS and HACE. However, there are other factors to be considered before discarding the theory of VEGF as a central mediator in this process. These considerations have been described above, but all rest on the assumption that current human studies lack the methodological sophistication necessary to adequately assess VEGF levels in the region of interest, namely the brain.

### Limitations

There are several limitations to this study. First, 21 of 72 climbers (29.2%) refused enrollment in the study. The lack of consecutive enrollment may have selected for healthier subjects, i.e., those less likely to have symptoms of AMS. Second, our dependence on solar energy to power the centrifuge meant that subjects could only be enrolled during periods of sunny weather. Due to the unstable weather during the 2002 climbing season, 16 of 25 days (64%) were partially or completely unsuitable for subject enrolment. Third, despite an attempt to standardize the documentation of the symptomatology, self-reporting of AMS symptoms is subjective and, given the small study population, the findings may not be generalizabile. Finally, given the large range, and thus standard deviation, of serum VEGF in healthy subjects at sea level,[13] it may be difficult to determine significance without a large study size or matched controls.

## CONCLUSION

This study does not support the hypothesis that serum VEGF levels correlate with symptoms of AMS.
